# Metronomic adjuvant chemotherapy evaluation in locally advanced head and neck cancers post radical chemoradiation – a randomised trial

**DOI:** 10.1016/j.lansea.2023.100162

**Published:** 2023-02-24

**Authors:** Vijay Patil, Vanita Noronha, Nandini Menon, Vijayalakshmi Mathrudev, Atanu Bhattacharjee, Kavita Nawale, Deevyashali Parekh, Shripad Banavali, Kumar Prabhash

**Affiliations:** a Department of Medical Oncology, Tata Memorial Centre, HBNI, Mumbai, India; b Section of Biostatistics, Centre for Cancer Epidemiology, Tata Memorial Centre, HBNI, Mumbai, India

**Keywords:** Metronomic chemotherapy, Head and neck cancer, Squamous cell carcinoma, Maintenance

## Abstract

**Background:**

Locally advanced head and neck cancers treated with radical chemoradiation have unsatisfactory outcomes. Oral metronomic chemotherapy improves outcomes in comparison to maximum tolerated dose chemotherapy in the palliative setting. Limited evidence suggests that it may do so in an adjuvant setting. Hence this randomized study was conducted.

**Methods:**

Patients of head and neck (HN) cancer with primary in oropharynx, larynx or hypopharynx, with PS 0–2 post radical chemoradiation with documented complete response were randomized 1:1 to either observation or oral metronomic adjuvant chemotherapy (MAC) for 18 months. MAC consisted of weekly oral methotrexate (15 mg/m^2^) and celecoxib (200 mg PO BD). The primary endpoint was OS and the overall sample size was 1038. The study had 3 planned interim analyses for efficacy and futility. Trial registration- Clinical Trials Registry- India (CTRI): CTRI/2016/09/007315 [Registered on: 28/09/2016] Trial Registered Prospectively.

**Findings:**

137 patients were recruited and an interim analysis was done. The 3 year PFS was 68.7% (95% CI 55.1–79.0) versus 60.8% (95% CI 47.9–71.4) in the observation and metronomic arm respectively (P value = 0.230). The hazard ratio was 1.42 (95% CI 0.80–2.51; P value = 0.231). The 3 year OS was 79.4% (95% CI 66.3–87.9) versus 62.4% (95% CI 49.5–72.8) in the observation and metronomic arm respectively (P value = 0.047). The hazard ratio was 1.83 (95% CI 1.0–3.36; P value = 0.051).

**Interpretation:**

In this phase 3 randomized study, oral metronomic combinations of weekly methotrexate and daily celecoxib failed to improve the PFS or OS. Hence observation post-complete response post radical chemoradiation remains the standard of care.

**Funding:**

ICON funded this study.

**Research in context**

**Evidence before this study**

A pubmed search was performed at the time of conceptualisation of the study with keywords,“metronomic chemotherapy”, “Maintenance chemotherapy”, “Head and neck carcinoma” and “head and neck squamous cell carcinoma”. All articles from 1st January 1990 till 30th May 2016 were included. We failed to identify any prospective data regarding use of metronomic chemotherapy post definitive chemoradiation. However we had published a retrospective analysis of adjuvant metronomic chemotherapy in patients who had received adjuvant radiation or chemoradiation in oral squamous cell carcinoma. We had observed an absolute improvement in disease free survival by 14.9%. Similar promising results regarding the use of metronomic regimen with oral UFUR and S1 were reported by Lin et al. and Furusaka et al. Considering all three studies had reported very limited adverse events and had shown promising improvement in outcomes. We decided to undertake this phase 3 randomized study in locally advanced head and neck squamous cell carcinoma where patients were randomized post definitive chemoradiation to either observation or adjuvant metronomic chemotherapy.

**Added value of this study**

137 patients were recruited and an interim analysis was done. The 3 year progression free survival (PFS) was 68.7% (95% CI 55.1–79) versus 60.8% (95% CI 47.9–71.4) in the observation and metronomic arm respectively (P value = 0.230). The hazard ratio was 1.42 (95% CI 0.80–2.51; P value = 0.231). The 3 year overall survival (OS) was 79.4% (95% CI 66.3–87.9) versus 62.4% (95% CI 49.5–72.8) in the observation and metronomic arm respectively (P value = 0.047). The hazard ratio was 1.83 (95% CI 1.0–3.36; P value = 0.051). Thus study suggested that addition of metronomic chemotherapy led to a decrease in PFS and OS.

**Implications of all the available evidence**

We have reported in addition to this study, two more randomized studies which had similar findings. One in recurrent HNSCC who had undergone salvage surgery and had high risk features but were unsuitable of Re-RT. These patients were randomized to observation versus metronomic chemotherapy. This study also suggested that metronomic chemotherapy not only failed to improve outcomes but had inferior outcomes. Similar results were seen in another study performed in squamous cell carcinoma esophagus post chemoradiation of adjuvant metronomic chemotherapy. The metronomic regimen used was a combination of methotrexate and celecoxib in all 3 studies. Considering all available evidence, metronomic chemotherapy combination of methotrexate and celecoxib should not be recommended as adjuvant therapy in HNSCC post definitive chemoradiation who had achieved complete response.

## Introduction

Head and neck squamous cell carcinomas (HNSCC) are one of the commonest tumors in India.^1^ The sites of lip & oral cavity, larynx, hypopharynx and oropharynx contribute 135,929, 34,687, 28,489 and 20,617 cancer patients each year in India.^1^ Unfortunately >80% HNSCC patients present in the locally advanced stage.^2^ In locally advanced stages HNSCC have poor survival rates with 2, 5 and 10 years overall survival (OS) post standard fractionation radiation being 45.6%, 29.3% and 18.3% respectively.^3^ The addition of concurrent chemotherapy to radiation leads to an improvement in survival. The 2 and 5-year overall survival post concurrent chemoradiation are 55.0% and 33.7% in accordance with the MACH-NC analysis.^4^ Even when chemoradiation is done for larynx preservation the overall survivals are, at the best, modest. The results of RTOG 99–11 revealed a 5-year and 10-year overall survival rate of 55.1% and 27.5%.^5^

The metronomic combination of methotrexate and celecoxib is a tolerable regimen with minimal grade 3-4 adverse events and has proven efficacious over cisplatin in the palliative setting.^6^ In palliative setting in a large randomized study of 422 patients use of metronomic chemotherapy regimen of methotrexate and celecoxib had an overall survival advantage over intravenously administered cisplatin (unadjusted hazard ratio for death 0.773 [95% CI 0.615–0.97, p = 0.026]). Use of metronomic chemotherapy also decreased the rate of grade 3 or above adverse events from 30% to 19% (P = 0.01). In advanced solid tumors, it's proposed that adjuvant treatment with metronomic chemotherapy in preclinical mouse models limits metastatic spread.^4^, ^7^, ^8^ Multiple retrospective and prospective studies have suggested that adjuvant metronomic chemotherapy may improve outcomes in HNSCC.^9^, ^10^, ^11^, ^12^, ^13^ In view of the encouraging data from palliative setting and small clinical data in the adjuvant setting we decided to test it in the adjuvant setting. Hence we did a phase 3 randomized study to assess whether adjuvant treatment with the metronomic combination of methotrexate and celecoxib leads to improvement in outcome in locally advanced head and neck cancers post radical chemoradiation. The null hypothesis was that adjuvant metronomic chemotherapy will not result in an absolute improvement in 3-year overall survival rate.

## Methods

### Patients

Adult patients (≥18 years), Eastern Cooperative Oncology Group (ECOG) performance status (PS) 0–2, locally advanced head and neck squamous cell carcinoma (HNSCC), primary in pharynx (oropharynx or hypopharynx) or larynx (supraglottis or glottis or subglottis), normal organ function and with complete response post radical chemoradiation were included in the study. The complete response was defined as per Response Evaluation Criteria in Solid Tumors (RECIST) version 1.1 and modalities used were either Positron emission tomography- Computed tomography (PET-CT) or contrast enhanced CT scan. Patients with uncontrolled comorbidities or 6 months post completion of chemoradiation (CTRT) or pregnant or breastfeeding females were excluded.

### Trial design and procedures

This was a 2 arm, parallel design, open-label, superiority, explanatory phase III randomized controlled trial with multiple interim analyses. Stratified randomization was performed. The stratification factors were the site of the tumor (oropharynx versus larynx versus hypopharynx), T-grouping (T1-2, T3, T4) and N-grouping (N0, N1, N2–N3). The patients randomized in metronomic adjuvant chemotherapy (MAC) arm were self-administered oral methotrexate 15 mg/m2 weekly (Day 1, 8, 15 and 22) every 28-day cycle while celecoxib was self-administered oral 200 mg twice daily. The combination was continued to a maximum of 18 such cycles; unless prohibitive toxicity or disease progression occurred prior to it. Patients in the observation arm were kept on follow-up only. The follow-up in both arms was done till death.

### Endpoints and definition

The primary endpoint was 3-year overall survival (OS). OS was defined time between the date of randomization to the date of death or date of last follow up whichever was applicable. Patients alive at their last follow-ups were censored. The other key secondary endpoints were 3-year progression-free survival (PFS), adverse events and quality of life. PFS was defined as the duration of time between the date of randomization to the date of progression or death whichever was earlier. Patients who have not progressed or died at their last follow-ups were censored. Adverse events were documented in accordance with Common Terminology Criteria for Adverse Events version 4 (CTCAE version 4.03).

### Sample size calculation

The 3 years overall survival post radical CTRT in PET-CT negative patients was assumed to be 69%. With type I error of 5%, Type II error of 20%, for an improvement in a hazard ratio to 0.77, with a study duration of recruitment of 9 years, study follow-up of 3 years and 5% lost to follow up the sample size was calculated using fixed design, two-arm trial with the time-to-event outcome (Lachin and Foulkes, 1986). However, in view of the large sample size and limited data availability for metronomic adjuvant, 3 interim analyses were planned in this study using group sequential design sample size with Hwang-Shih-DeCani spending function for time-to-event outcome events. The final sample size was 1038 patients with the final analysis done at 530 events. The planned interim analysis was at 133, 265 and at 398 events.

### Oversight

The study protocol was approved by the institutional ethics committee and the study was registered with the Clinical Trials Registry of India (CTRI)-CTRI/2016/09/007315 on 28th September 2016. The study was conducted in accordance with norms set by the Declaration of Helsinki, the International Council for Harmonisation (ICH)- Good Clinical Practice (GCP) and the Indian Council of Medical Research (ICMR). The study was monitored by an independent institutional data monitoring and safety board. The study was stopped early and analysis was performed because of two reasons. Firstly the study recruited slowly, and the recruitment stopped during the Coronavirus disease (COVID-19) pandemic. Secondly, two similar studies of metronomic adjuvant chemotherapy in 2021, one in squamous cell carcinoma esophagus^14^ and one post salvage surgery in HNSCC^15^ were not only negative but suggested harm from metronomic chemotherapy. Thus the decision was taken by the Head and neck disease management group to stop the study and IEC was informed about the same. As the concern was patient safety and the authors had no intention of continuing the study the study was stopped and analysis was performed. The trigger for analysis was patient safety.

### Statistical analysis

The 3 year OS and PFS were estimated using the Kaplan Meier method and compared between the 2 arms by the log-rank test. Cox proportional hazard model was constructed for the calculation of the hazard ratio. The model was used to see the impact of chemotherapy regimen on 3 year OS and PFS in accordance with known prognostic factors. These were age (below or above 70 years), subsite, T grouping, N grouping, hemoglobin level (equal to or below 10 g/dl or above it), and ECOG PS (0 versus 1). The proportional hazards (PH) assumption was checked and met for PFS but not for OS analysis. Hence the restricted mean survival analysis was used for further OS analysis. The median follow-up was estimated using the reverse Kaplan Meier technique. The worst grade toxicity between the 2 arms was compared using the Fisher exact test. A p-value of 0.05 was considered significant. The data were censored for analysis on 22nd May 2021.

### Role of funding source

The funding agency had no role in the design, conduct of the study, collection, management, analysis, and interpretation of the data, preparation, review or approval of the manuscript, and decision to submit the manuscript for publication.

## Results

### Baseline characteristics

**Fig. 1:**
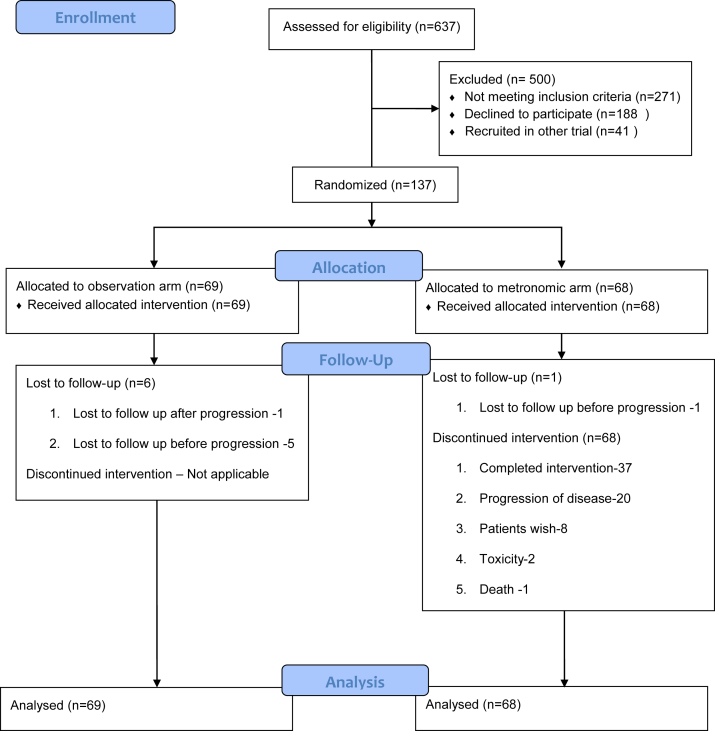
CONSORT diagram.

The study enrolled 137 patients between 3rd November 2016 and 20th August 2019 and Consolidated Standards of Reporting Trials (CONSORT diagram) (Fig. 1) depicts the flow of the patient. The baseline characteristics were well matched between the 2 groups and are shown in Table 1.

**Table 1:** Baseline characteristics at the time of randomisation.

Variable	Observation arm (n = 69)	Metronomic arm (n = 68)
Age		
Median (Range)	58 (29–73)	57.5 (26–74)
Elderly-No (%)	32 (46.4)	31 (45.6)
Gender-No (%)		
Male	61 (88.4)	65 (95.6)
Female	8 (11.6)	3 (4.4)
ECOG PS-No (%)		
0	3 (4.3)	3 (4.4)
1	66 (95.7)	65 (95.6)
Site-No (%)		
Oropharynx	29 (42.0)	30 (44.1)
Larynx	22 (31.9)	20 (29.4)
Hypopharynx	18 (26.1)	18 (26.5)
T-Grouping-No (%)		
T1-T2	13 (18.8)	18 (26.5)
T3-T4	56 (81.2)	50 (73.5)
N-grouping-No (%)		
N0–N1	37 (53.6)	35 (51.5)
N2–N3	32 (46.4)	33 (48.5)
Stage-No (%)		
Stage III	26 (37.7)	23 (33.8)
Stage IVA	37 (53.6)	37 (54.4)
Stage IVB	6 (8.7)	8 (11.8)
Radiation technique-No (%)		
IMRT	20 (29.0)	19 (27.9)
3DCRT	47 (68.1)	43 (63.2)
Conventional	2 (2.9)	6 (8.8)
Concurrent-No (%)		
Cisplatin	57 (82.6)	57 (83.8)
Carboplatin	10 (14.5)	6 (8.8)
Others	2 (2.9)	5 (7.4)
Baseline hemoglobin level-No (%)		
= <10 g/dl	2 (2.9)	4 (5.9)
>10 g/dl	67 (97.1)	64 (94.1)

IMRT- Intensity-modulated radiotherapy, 3DCRT- 3D conformal radiation therapy, Eastern Cooperative Oncology Group (ECOG) performance status (PS) and g/dl-gram per deciliter. Elderly was defined as age 60 years or above.

### Outcomes

**Fig. 2:**
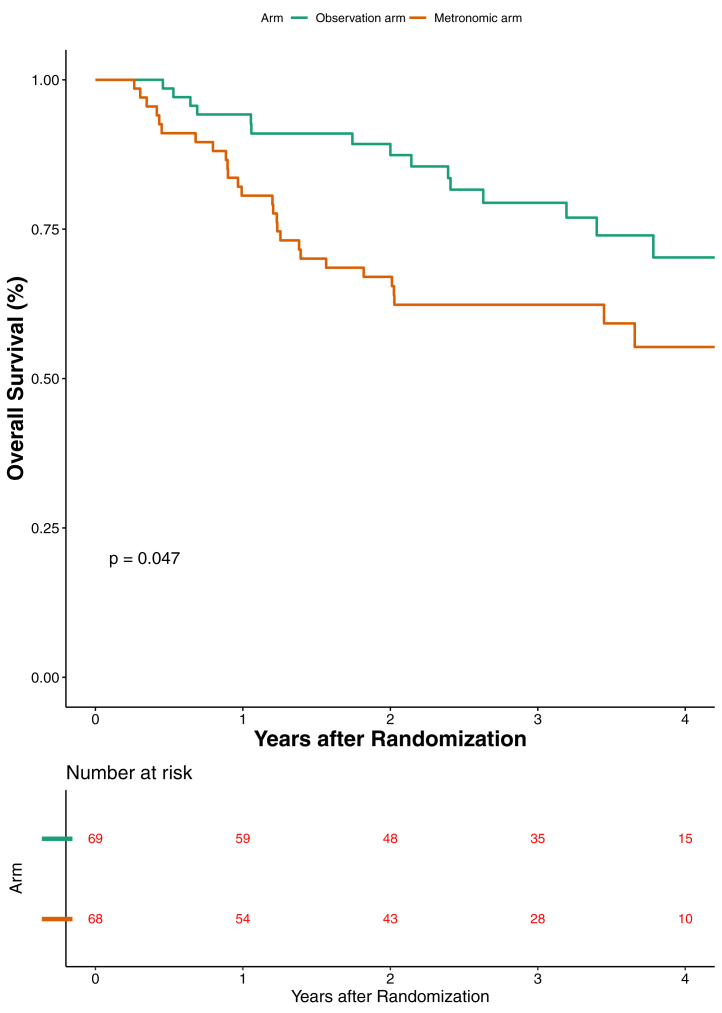
Overall survival (OS) graph. Elderly was defined as age 60 years or above. Eastern Cooperative Oncology Group (ECOG) performance status (PS) and g/dl-gram per deciliter.

The median follow up was 3.39 (IQR 2.15–4.13) in the observation arm versus 3.36 (IQR 2.64–4.03) years in the metronomic arm. The corresponding events of death in each arm were 17 and 27 respectively. The median overall survival was not reached (95% CI 4.31-NA) in the observation and was not reached (95% CI 2.03-NA) even in the metronomic arm (Fig. 2). The 3 year OS was 79.4% (95% CI 66.3–87.9) versus 62.4% (95% CI 49.5–72.8) in the observation and metronomic arm respectively (P value = 0.047). The hazard ratio was 1.83 (95% CI 1.0–3.36; P value = 0.051). However the proportional hazard assumption was violated and hence restricted mean analysis was performed. The restricted mean survival duration was 3.46 (95% CI 3.20–3.72) and 2.87 (95% CI 2.52–3.21) years in the observation and the metronomic arm respectively (Fig. S1). The difference in restricted mean survival duration was 0.59 (95% CI 0.16–1.02; P-value = 0.007) years. The estimated ratio of time lost due to administration of metronomic chemotherapy was 0.48 (95% CI 0.27–0.84; P-value = 0.011). This ratio quantifies the amount of time lost due to exposure to the metronomic arm. Restricted mean survival time (RMST) is defined as the area under the survival (AUC) curve up to a specific time point. The difference is the ratio of AUC of both arms. The most optimal outcome is 100% survival till the specific time point chosen. The AUC is RMST. The area above the curve up to the 100% mark excluding area covered under RMST is estimated time lost for each arm respectively. The ratio of these areas is the ratio of time lost. The cause of death in each arm is shown in Table S1. The impact of metronomic chemotherapy on OS in accordance with known prognostic factors is shown in Table S3.

**Fig. 3:**
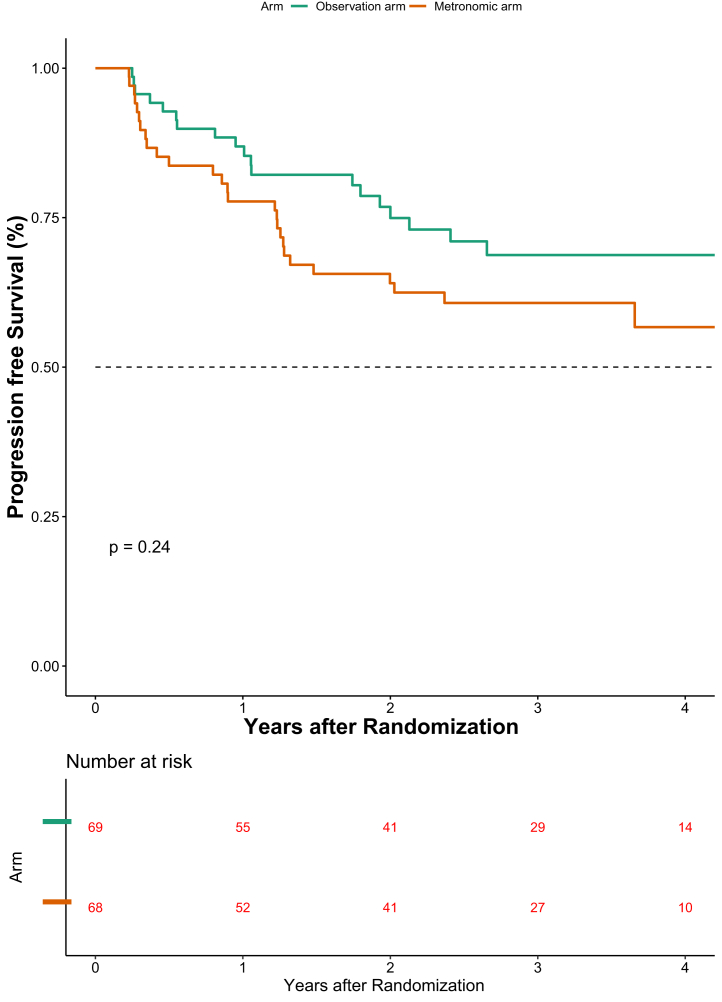
Progression free survival (PFS) graph. Elderly was defined as age 60 years or above. Eastern Cooperative Oncology Group (ECOG) performance status (PS) and g/dl-gram per deciliter.

The events for progression were seen in 21 and 27 patients in the observation and the metronomic arm respectively. The 3 year PFS was 68.7% (95% CI 55.1–79.0) versus 60.8% (95% CI 47.9–71.4) in the observation and metronomic arm respectively (P value = 0.230, Fig. 3). The hazard ratio was 1.42 (95% CI 0.80–2.51; P value = 0.231). The pattern of the first site of failure is shown in Table S2. The impact of metronomic chemotherapy on PFS in accordance with known prognostic factors is shown in Table S4.

### Adverse events and compliance

Oral metronomic chemotherapy was started in 68 patients. At the time of analysis, it was discontinued in all patients. The reasons for discontinuation were the completion of 18 cycles in 37, progression of disease in 20, patient choice in 8, intolerable adverse events in 2 and death in 1. The intolerable adverse event was mucositis in both patients. Dose reduction was required in 6 patients. It was because of mucositis in 5 patients and rise in SGOT & SGPT in 1 patient. Non-compliance to oral metronomic chemotherapy was seen in 2 patients, one due to COVID-19 lockdown and in another due to the patient's choice. Adverse events are depicted in Table 2.

**Table 2:** Adverse events between observation and metronomic arms.

Variable	Observation arm (n = 69)		Metronomic arm (n = 68)		P-value	
	Any grade	Grade 3−5	Any grade	Grade 3−5	Any grade	Grade 3−5
Anemia	28 (40.6)	–	31 (45.6)	1 (1.5)	0.607	0.496
Neutropenia	8 (11.6)	1 (1.4)	13 (19.1)	2 (2.9)	0.245	0.619
Thrombocytopenia	6 (8.7)	1 (1.4)	23 (30.3)	2 (2.9)	0.021	0.619
SGOT rise	2 (2.9)	–	12 (17.6)	2 (2.9)	0.005	0.245
SGPT rise	2 (2.9)	1 (1.4)	15 (22.1)	3 (4.4)	0.001	0.366
Hyponatremia	22 (31.9)	6 (8.7)	24 (35.3)	6 (8.8)	0.720	1
Hypokalemia	1 (1.4)	–	1 (1.5)	–	1.000	–
Hyperkalemia	2 (2.9)	–	7 (10.3)	1 (1.5)	0.097	0.496
Hypocalcemia	1 (1.4)	–	3 (4.4)	–	0.366	–
Hypercalcemia	8 (11.6)	–	1 (1.5)	–	0.033	–
Hypomagnesemia	4 (5.8)	–	8 (11.8)	–	0.243	–
Hypermagnesemia	3 (4.3)	–	2 (2.9)	–	1.00	–
Rash	–	–	3 (4.4)	–	0.120	–
Mucositis	18 (26.1)	–	32 (47.1)	6 (8.8)	0.013	0.013
Odynophagia	30 (43.5)	3 (4.3)	33 (48.5)	5 (7.4)	0.609	0.493

## Discussion

To the best of our knowledge, this is the first, phase 3 randomized study exploring the use of MAC post radical chemoradiation. Unfortunately, the study was negative and MAC failed to improve the PFS and OS. The results are surprising. In a palliative setting the same regimen as opposed to single-agent cisplatin had led to an improvement in PFS and OS. However, it is unclear why the same regimen, when given as an adjuvant, failed to improve outcomes. Interestingly even the PFS was numerically lower in the MAC arm and a similar finding was observed in the OS too. This suggests that the MAC biologically failed to decrease the rate of recurrence in locally advanced head and neck squamous cell carcinoma (LAHNSCC). The reason is probably related to the mechanism of action of metronomic chemotherapy. Metronomic therapy's mechanisms of action, whether as antiangiogenic or as immunomodulators, are both related to the modification of the tumor microenvironment. Probably in the absence of macroscopic tumor residue, as only patients with complete response were included, metronomic chemotherapy failed to act. This might also be the reason for the negative results of other MAC studies which prompted an early analysis. However we have at present no explanation why the disease recurred early in the MAC arm.

LAHNSCC except Human Papillomavirus (HPV)-positive oropharyngeal cancer has modest outcomes.^16^ Especially LAHNSCC seen in the Indian subcontinent, even if they have a primary in the oropharynx, are largely HPV negative and are predominantly related to tobacco hence have poor outcomes.^16^^,^^17^ Strategies to improve upon these outcomes of concurrent chemoradiation with the use of neoadjuvant chemotherapy^18^, ^19^, ^20^ or the use of altered fractionated radiation^21^ or the addition of additional radiosensitizer to cisplatin^22^ have largely been unsuccessful. Trials to improve these outcomes with the addition of checkpoint inhibitors are ongoing. However, the results of the addition of avelumab to cisplatin and radiation were presented at ESMO 2020 and unfortunately, it failed to improve outcomes.^23^ Hence, at present Cisplatin-Radiation remains the cornerstone of management in these patients.

In this study, the LAHNSCC included those who had a complete response post-CTRT. Patients who have a complete response post-CTRT have a better prognosis than those patients who fail to achieve a complete response. Thus the cohort of patients selected in this study was a good prognostic cohort and improving outcomes further in this cohort might be challenging. This was done as administration of methotrexate, celecoxib during CTRT and the immediate post-CTRT period was considered a challenge. The tolerance of methotrexate, celecoxib and cisplatin co-administration has never been studied before and was theoretically considered an intolerable regimen. Secondly, the oral administration of these drugs during the 4-7th week of CTRT and immediately post-CTRT was considered difficult as most patients would have dysphagia and would require a feeding tube. Hence the investigators contemplated waiting till at least 3 months post-CTRT completion for adverse events to resolve. Further 3 months post-CTRT, we routinely perform response assessments and hence this was a logical choice. Patients with residual disease at first response assessment are subjected to either salvage surgery or palliative chemotherapy and are not observed hence these patients were excluded.

MAC had side effects. These were mainly grade 1-2 adverse events. Grade 3 or above adverse events were seen in very few patients, thus attesting to the safety of this regimen. Thus this regimen can be further modified to make it effective. At the time of planning this study a 2 drug metronomic of oral methotrexate (15 mg/m2) and the celecoxib (200 mg PO BD) was used in our institute. Hence the study was planned with these drugs in the above mentioned doses. Now subsequently we have refined this regimen and are using a triple drug metronomic where we have added erlotinib 150 mg daily and have decreased the dose of methotrexate from 15 mg/m2 to 9 mg/m2. A triple metronomic regimen has a higher activity than a double metronomic regimen.^24^ The response rate of the triple metronomic regimen is 45.3% (95% CI, 34.6–56.6)^25^ as opposed to double metronomic regimen of 13.1 (9.23–18.42).^6^ However further studies are required to see whether this triple metronomic regimen can be effective as adjuvant therapy. It might be worthwhile to explore the triple metronomic regimen as even if future trials report an improvement in outcome with the addition of checkpoint inhibitors, this regimen would be financially inaccessible to a large majority of the global population.

The study has its own limitations and strengths. HPV testing was not done in all oropharyngeal patients. As the study was conducted in a time period where it was not yet adopted as routine practice at the study site. However >90% of oropharyngeal patients from the author institute are HPV negative. The study did not complete recruitment and hence results need to be interpreted with caution. However, the results are unlikely to be changed if the recruitment was completed. The study was performed in a single center. However, it was performed in a premier cancer center in India, which registers over 10 000 new cases of HNSCC every year from all over India. So, although single-center, the study recruited from multiple states of India which are larger than multiple countries across the world. The strength of the study was it was performed using oral drugs, with compliance upwards of 80% and with a loss to follow-up rate of below 10%.

In this phase 3 randomized study, oral metronomic combinations of weekly methotrexate and daily celecoxib failed to improve the PFS or OS. Hence observation post-complete response post radical chemoradiation remains the standard of care.

## Contributors

The authors confirm contribution to the paper as follows:

Study conception and design: VP, KP.

Data collection: All authors.

Analysis and interpretation of results: All authors.

Draft manuscript preparation: All authors.

All authors reviewed the results and approved the final version of the manuscript. All authors vouch for the accuracy and completeness of the data, analyses and for the fidelity of the study to the study protocol.

## Data sharing statement

De-identified data may be shared on a case-by-case basis upon reasonable requests to the corresponding author.

## Declaration of interests

**Vijay Maruti Patil receivedfunding from**10.13039/100004325AstraZeneca (Inst); 10.13039/501100003769Eisai Germany (Inst); Intas (Inst); 10.13039/100004331Johnson & Johnson/Janssen (Inst); NATCO Pharma (Inst); 10.13039/100004336Novartis (Inst).

**Vanita Noronha receivedfunding from**10.13039/100002429Amgen (Inst); 10.13039/100004325AstraZeneca (Inst); Dr. Reddy's Laboratories (Inst); Intas (Inst); Sanofi/Aventis (Inst).

**Kumar Prabhash receivedfunding from**Alkem Laboratories (Inst); BDR Pharmaceutics (Inst); 10.13039/100007777Biocon (Inst); Dr. Reddy's Laboratories (Inst); Fresenius Kabi (Inst); NATCO Pharma (Inst); Roche (Inst).

## Appendix A Supplementary data

Supplementary appendix

Metronomic adjuvant _ Post CTRT
