# Supplementary material for: Metronomic adjuvant chemotherapy evaluation in locally advanced head and neck cancers post radical chemoradiation – a randomised trial
Source: Lancet Reg Health Southeast Asia. 2023 Feb 24;12:100162. doi: 10.1016/j.lansea.2023.100162 (PMC10305911; doi:10.1016/j.lansea.2023.100162)
Supplement: Supplementary appendix [file mmc1.docx]

Supplementary appendix

[**Table S1**](#_ur31fq42w13r) **2**

[**Table S2**](#_tvvuyk9ihf5p) **3**

[**Table S3**](#_wp5695ec33io) **4**

[**Table S4**](#_vtr5is7q8dki) **7**

[**Figure S1**](#_u73locsbz9lf) **10**

# Table S1

| Variable | Observation arm (n=69) | Metronomic arm (n=68) |
| --- | --- | --- |
| Death due to any cause-No(%) | 17(24.6) | 27(39.7) |
| Death due progressive disease-No(%) | 13(18.8) | 25(36.8) |
| Death due to other causes-No(%) | 1(1.4) | 1(1.5) |
| Death due to unknown reasons-No(%) | 3(4.3) | 1(1.5) |

Table S1- Table depicting the causes of death in each arm.

# Table S2

| Variable | Observation arm (n=69) | Metronomic arm (n=68) |
| --- | --- | --- |
| Local failure-No(%) | 10(14.5) | 16(23.5) |
| Regional failure-No(%) | 11(15.9) | 9(13.2) |
| Distant failure-No(%) | 1(1.4) | 7(10.3) |

Table S2- Table depicting the first site of failure pattern in each arm. A few patients had more than 1 site of first failure.

# Table S3

| Variable | | Arm | N | OS event | 3 year OS | HR with 95% CI | P-Value |
| --- | --- | --- | --- | --- | --- | --- | --- |
| Age | Non Elderly | Observation | 37 | 8 | 80.4(61-90.8) | 2.118(0.887-5.054) | 0.091 |
|  |  | MAC | 37 | 15 | 61.4(43.6-75.1) |  |  |
|  | Elderly | Observation | 32 | 9 | 78.8 (58.6-90) | 1.584(0.666-3769) | 0.298 |
|  |  | MAC | 31 | 12 | 63.3(43.6-77.8) |  |  |
| ECOG PS | PS 0 | Observation | 3 | 1 | 66.7(5.41-94.5) | 1.405(0.125-15.84) | 0.783 |
|  |  | MAC | 3 | 2 | 33.3(0.8-77.4) |  |  |
|  | PS 1 | Observation | 66 | 16 | 80.4(67.1-88.7) | 1.825(0.973-3.421) | 0.061 |
|  |  | MAC | 65 | 25 | 63.8(50.7-74.3) |  |  |
| T grouping | T0-T2 | Observation | 13 | 3 | 83.1(47.2-95.5) | 3.714(1.016-13.57) | 0.047 |
|  |  | MAC | 18 | 10 | 47.1(23-68) |  |  |
|  | T3-T4 | Observation | 56 | 14 | 78.6(63.4-88) | 1.381(0.68-2.804) | 0.372 |
|  |  | MAC | 50 | 17 | 67.6(52.7-78.8) |  |  |
| N grouping | N0-N1 | Observation | 37 | 3 | 94.6(80.1-96.7) | 1.988(0.496-7.967) | 0.332 |
|  |  | MAC | 35 | 6 | 85.7(69-93.8) |  |  |
|  | N2-N3 | Observation | 32 | 14 | 65.7(45.2-80) | 2.317(1.169-4.592) | 0.016 |
|  |  | MAC | 33 | 21 | 35.7(19.5-52.4) |  |  |
| Hemoglobin | =<10 g/dl | Observation | 2 | 0 | 100 | 1.04 X109 (0-Infinity) | 0.999 |
|  |  | MAC | 4 | 2 | 50(5.8-84.5) |  |  |
|  | >10 g/dl | Observation | 67 | 17 | 78.7(65.3-87.4) | 1.742(0.94-3.23) | 0.078 |
|  |  | MAC | 64 | 25 | 63.1(49.9-73.8) |  |  |
| Site | Oropharynx | Observation | 29 | 10 | 77.4(56.1-89.3) | 1.786(0.79-4.03) | 0.163 |
|  |  | MAC | 30 | 14 | 51.6(32.3-67.8) |  |  |
|  | Hypopharynx | Observation | 18 | 6 | 65.8(35.9-84.3) | 1.842 (0.666-5.097) | 0.239 |
|  |  | MAC | 18 | 10 | 48.1(23.9-68.9) |  |  |
|  | Larynx | Observation | 22 | 1 | 92.9(59.1-99.0) | 3.138(0.326-30.21) | 0.322 |
|  |  | MAC | 20 | 3 | 90.0(65.6-97.4) |  |  |

Table S3- Table depicting impact of metronomic chemotherapy in each subgroup on OS. Elderly was defined as age 60 years or above.Eastern Cooperative Oncology Group (ECOG) performance status (PS) and g/dl- gram per deciliter.

#

# Table S4

| Variable | | Arm | N | PFS event | 3 year PFS | HR with 95% CI | P-Value |
| --- | --- | --- | --- | --- | --- | --- | --- |
| Age | Non Elderly | Observation | 37 | 10 | 72.9(53.8-85.1) | 1.667(0.742-3.744) | 0.216 |
|  |  | MAC | 37 | 15 | 58.5(40.7-72.6) |  |  |
|  | Elderly | Observation | 32 | 11 | 65.2(44.7-79.7) | 1.198(0.538-2.721) | 0.666 |
|  |  | MAC | 31 | 12 | 63.5(43.8-77.9) |  |  |
| ECOG PS | PS 0 | Observation | 3 | 1 | 66.7(5.41-94.52) | 1.405(0.125-15.84) | 0.783 |
|  |  | MAC | 3 | 2 | 33.3(0.8-77.4) |  |  |
|  | PS 1 | Observation | 66 | 20 | 69.2(55.3-79.6) | 1.388(0.770-2.5) | 0.276 |
|  |  | MAC | 65 | 25 | 62.1(48.9-72.8) |  |  |
| T grouping | T0-T2 | Observation | 13 | 3 | 84.6(51.2-95.9) | 3.768(1.029-13.79) | 0.045 |
|  |  | MAC | 18 | 10 | 47.4(23.2-68.3) |  |  |
|  | T3-T4 | Observation | 56 | 18 | 64.8(48.9-76.8) | 1.020(0.525-1.983) | 0.953 |
|  |  | MAC | 50 | 17 | 65.4(50.3-76.9) |  |  |
| N grouping | N0-N1 | Observation | 37 | 5 | 84.1(64.9-93.3) | 1.184(0.361-3.889) | 0.781 |
|  |  | MAC | 35 | 6 | 82.5(65.1-91.8) |  |  |
|  | N2-N3 | Observation | 32 | 16 | 54.9(35.9-70.4) | 1.831(0.951-3.528) | 0.071 |
|  |  | MAC | 33 | 21 | 36.2(19.9-52.8) |  |  |
| Hemoglobin | =<10 g/dl | Observation | 2 | 1 | 0 | 1.104(0.1-12.24) | 0.936 |
|  |  | MAC | 4 | 2 | 50(5.8-84.5) |  |  |
|  | >10 g/dl | Observation | 67 | 20 | 70(56.4-80.1) | 1.412(0.784-2.544) | 0.251 |
|  |  | MAC | 64 | 25 | 61.4(48.1-72.3) |  |  |
| Site | Oropharynx | Observation | 29 | 10 | 66.7(45.5-81.2) | 1.587(0.703-3.584) | 0.266 |
|  |  | MAC | 30 | 14 | 51.9(32.7-68.1) |  |  |
|  | Hypopharynx | Observation | 18 | 8 | 55(26.7-76.3) | 1.35(0.531-3.434) | 0.528 |
|  |  | MAC | 18 | 10 | 41.6(18.5-63.3) |  |  |
|  | Larynx | Observation | 22 | 3 | 81.6(52.4-93.8) | 1.036(0.194-4.807) | 0.966 |
|  |  | MAC | 20 | 3 | 90.0(65.6-97.4) |  |  |

Table S4- Table depicting impact of metronomic chemotherapy in each subgroup on PFS

#

# Figure S1


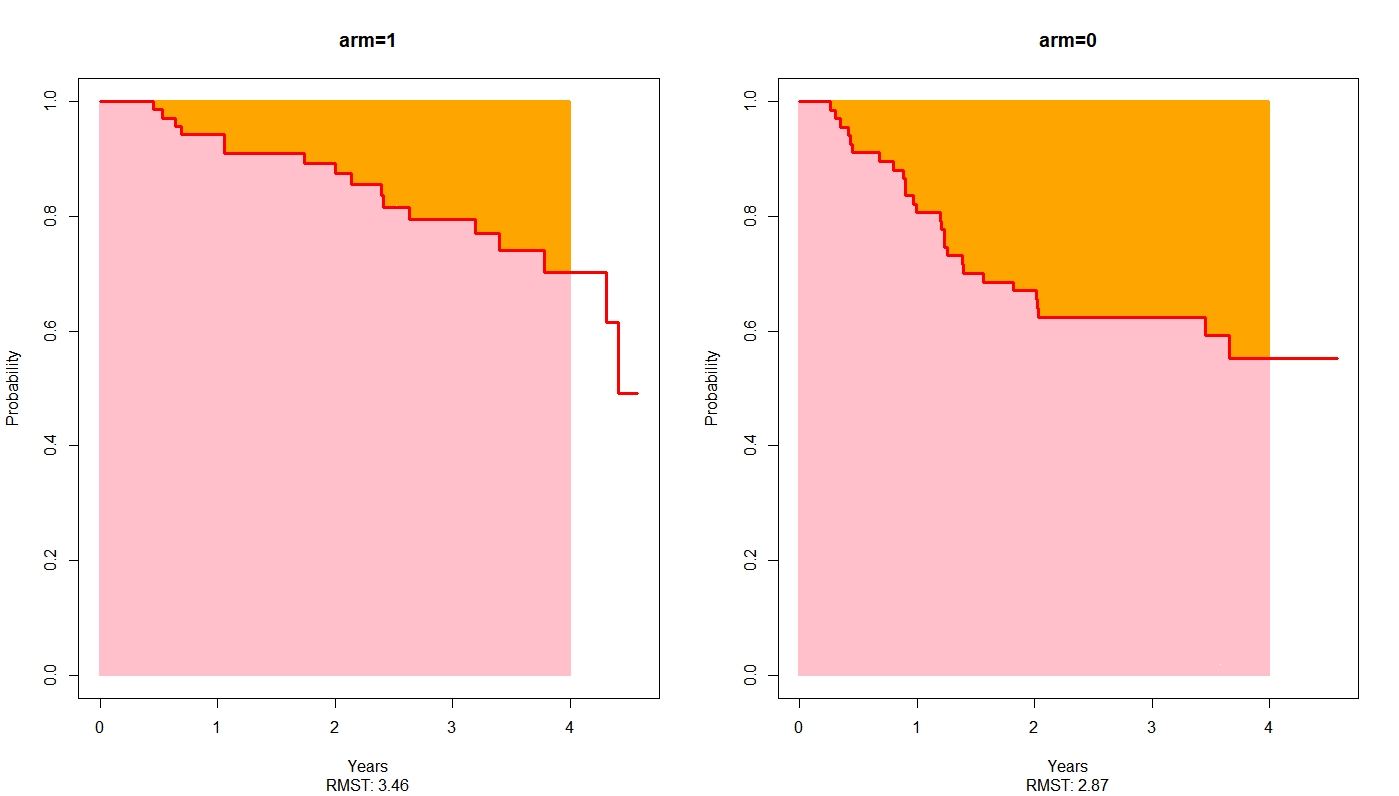


Figure S1 showing the restricted mean survival duration. Arm 1 depicts the observational arm, Arm 0 depicts the metronomic arm
