# Supplementary material for: Metronomic adjuvant chemotherapy evaluation in locally advanced head and neck cancers post radical chemoradiation – a randomised trial
Source: Lancet Reg Health Southeast Asia. 2023 Feb 24;12:100162. doi: 10.1016/j.lansea.2023.100162 (PMC10305911; doi:10.1016/j.lansea.2023.100162)
Supplement: Metronomic adjuvant _ Post CTRT [file mmc2.docx]

MACE-CTRT : Metronomic Adjuvant Chemotherapy Evaluation in locally advanced head and neck cancers post radical chemoradiation

Clinical trial protocol

Version 3.0

PI : Dr Vijay M Patil

# 1 Table of Contents

[1 Table of Contents](#_1uqllpnw4q57)

[2 Schema of the protocol](#_541qw0mgquvr)

[3 Concept sheet](#_2pfhpp5epleb)

[4 Background](#_6l97ouww4ub1)

[4.1 Outcomes post chemoradiation in locally advanced head & neck cancers](#_4b0stg5fjf1)

[4.2 Failure pattern post radical chemoradiation](#_ff4py6h6ut1k)

[4.3 Ineffective efforts & need for a new strategy](#_qsej89vckzu9)

[4.4 In Vivo data for metronomic](#_4envkab826ah)

[4.5 Metronomic chemotherapy : Clinical efficacy data in adjuvant setting](#_yvjh2dp4quye)

[4.6 Toxicity profile of metronomic therapy when used as adjuvant](#_y0s2peokkt8q)

[5 Rationale for the study](#_jdhxxx4yh7u0)

[6 Aims & Objectives](#_gqcnsvg0v9s4)

[6.1 Aim](#_xcth44df6cr7)

[6.2 Objective](#_9t11q9rw5u36)

[6.2.1 Primary objective](#_42asiity6v9b)

[6.2.2 Secondary objectives](#_yl3waasat6i0)

[7 Hypothesis & trial design](#_vvba3r6vov5c)

[8 Study setting](#_ox3jzdm3ssbo)

[9 Eligibility criteria](#_c1b5tlnvk7za)

[9.1 Inclusion criteria](#_rzl640cv6jm)

[9.2 Exclusion Criteria](#_btkaz8w4pp48)

[10 Interventions](#_vh0rirwcpruu)

[10.1 Registration](#_ij1wk83q6zoe)

[10.2 Arm A](#_jko1han7drfc)

[10.3 Arm B](#_aqk260ra442k)

[10.3.1 Schedule](#_ean69gzhgxt1)

[10.3.2 Administration](#_ar7yzy5rcpwc)

[10.3.3 Dose modifications](#_aojovaghfejr)

[10.3.3.1 Dose modification for hematological toxicity](#_gj1vlf1waknt)

[10.3.3.2 Dose modifications for other adverse events](#_6wnwc2izbhs)

[10.3.4 Start of a new cycle](#_jk4vxv2jb6q5)

[10.3.5 Concomitant Medications/Treatments](#_9fy285ctuo7z)

[10.3.5.1 Recommended](#_1w9m7dzjy0g)

[10.3.5.2 Permitted](#_hclu71c429nq)

[10.3.5.3 Use with caution](#_hvtjfgdzice1)

[10.3.5.4 Prohibited](#_1i8j3nm2bi17)

[10.3.6 Concomitant medication reporting](#_3mzn4oo7ke27)

[10.3.7 Compliance](#_71yparp5r6ir)

[10.4 Treatment discontinuation](#_mkxkiof4gu7n)

[10.5 Subsequent treatment](#_bcvfkg9bnw7j)

[11 Outcomes & Statistical measures](#_6zjqutebvyiz)

[11.1 Primary outcome](#_jt72kswfrkgf)

[11.2 Secondary outcomes](#_c4nbpgs5mfce)

[12 Participant timelines](#_r1faght95z6q)

[13 Sample size](#_1tksmuxcjzpd)

[13.1 Rationale for selection of primary endpoint : 3 year OS](#_visy44m7cqfl)

[13.2 Rationale for interim analysis](#_3xo6irlqasrd)

[13.3 Sample size justification](#_p3hll2xzbtuq)

[13.3.1 Sample size](#_e4fa6hx6qthw)

[13.3.2 Decision rules](#_ko90k3w3w2q3)

[14 Recruitment](#_16856da29ryu)

[15 Allocation of arm](#_v7sshtoqtkoi)

[15.1 Sequence generation](#_j2yny0t6fq26)

[15.2 Allocation concealment mechanism](#_ry9stzfjxbvo)

[15.3 Implementation](#_53afxx2e9llc)

[16 Data collection methods](#_fz4m25aq4ero)

[17 Data monitoring](#_ff3jjwsp9yuo)

[18 Harms](#_ejeqhm63oz5x)

[19 Auditing](#_g8ihdkk6mox0)

[20 Research ethics approval](#_lpns2rthkbzj)

[21 Protocol amendments](#_a7soc0i6ea5y)

[22 Consent](#_uviom9hroxb8)

[23 Confidentiality](#_uaerahynymq3)

[24 Access to data](#_a1yabd3zg59e)

[25 Ancillary & post trial care](#_cn1thdrr32lb)

[26 Dissemination policy](#_3mvilek7bfu)

[27 References](#_1d1a3vjfg4d1)

# 2 Schema of the protocol


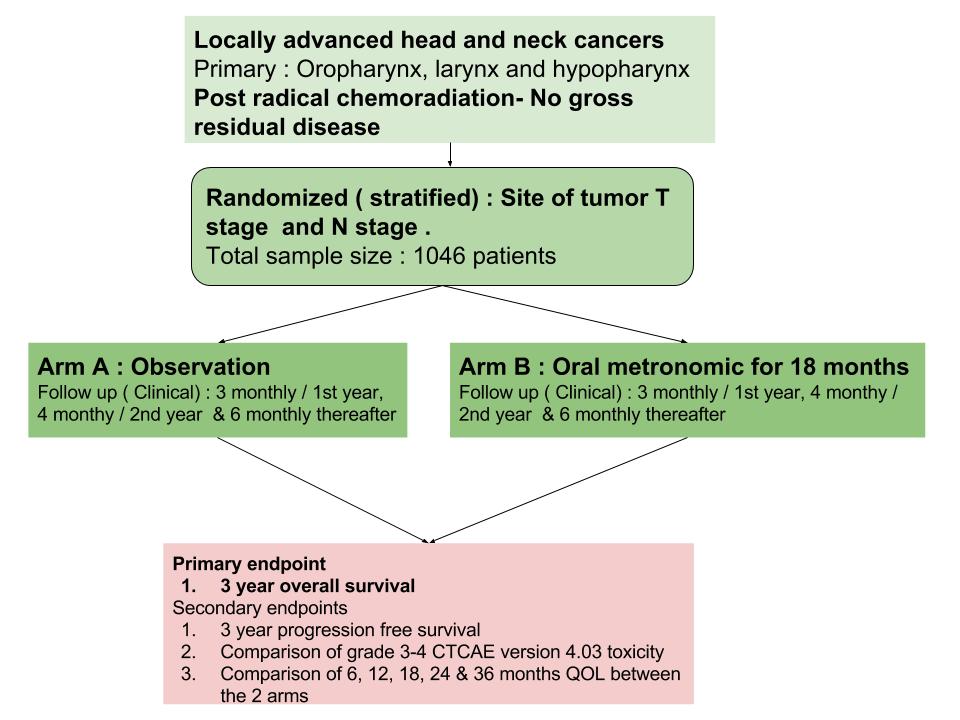


# 3 Concept sheet

| Primary Registry | Clinical Trial Registry of India (CTRI) |
| --- | --- |
| Contact for Public & scientific Queries | Dr Vijay M Patil, DM, Department of Medical Oncology, TMH  Email: vijaypgi@gmail.com |
| Scientific Title | MACE-CTRT : Metronomic Adjuvant Chemotherapy Evaluation in locally advanced head and neck cancers post radical chemoradiation |
| Countries of Recruitment | India |
| Health Condition Studied | Locally advanced head and neck cancers |
| Interventions | Arm A : Observation  Arm B : Oral metronomic chemotherapy for 18 months. Tab methotrexate 15 mg/m2 weekly PO and Capsule celecoxib 200 mg PO Twice daily |
| Aim | To study whether adjuvant treatment with metronomic chemotherapy leads to a betterment in outcome, in locally advanced head and neck cancers post radical chemoradiation |
| Objectives | Primary objective   1. To compare the overall survival at 3 years of adjuvant metronomic chemotherapy with that of observation in patients with locally advanced head and neck cancers post radical treatment   Secondary objectives   1. To compare the 3 year progression free survival of adjuvant metronomic chemotherapy with that of observation in patients with locally advanced head and neck cancers post radical treatment. . 2. To compare the grade 3-4 toxicity between adjuvant metronomic chemotherapy and observation 3. To compare the 6,12,18,24 & 36 months QOL score between the 2 arms |
| Population | **Key Inclusion criteria**   1. Participants must be post radical CTRT for a histologically confirmed squamous cell of head and neck region. 2. Participants must have no gross residual disease post 10-12 weeks of of completion of radical CTRT treatment . 3. Age : Any age above 18 years. No maximum age. 4. ECOG performance status ≤2 5. Participants must have normal organ and marrow function   **Key Exclusion criteria**   1. Participants who are receiving any other investigational agents. 2. Primary sites of malignancy major salivary gland or nasopharynx or skin 3. Patients with QTc prolongation defined as QTc interval greater than 480 ms |
| Study Type | 2 arm, parallel design, open label, superiority, explantory, integrated, sequential Phase3 with multiple interim , randomized controlled trial |
| Sample Size | Group sequential design sample size with 3 interim analysis for overall survival with sample size 1046. |
| Date of first enrollment | Post IRB approval and CTRI registration |
| Recruitment Status | Not opened |
| Outcome Measures | 1. 3 year OS : OS will be defined time in days between date of randomization to date of death. Patients alive at their last follow ups would be censored. The OS would be estimated by Kaplan meier analysis and would be compared between the 2 arms by the log rank test. Cox proportional hazard model would be constructed for calculation of hazard ratio. 2. 3 year PFS : PFS will be defined time in days between date of randomization to date of progression or death whichever is earlier. Patients who have not progressed or died at their last follow ups would be censored.The median PFS would be estimated by Kaplan meier analysis and would be compared between the 2 arms by the log rank test. Cox proportional hazard model would be constructed for calculation of hazard ratio. 3. Toxicity : To compare CTCAE version 4.03 worst grade toxicity between the 2 arms during any period of chemotherapy. Chi- square test or fisher's test would be used for comparison. The NCI Common Terminology Criteria for Adverse Events version 4 (NCI CTCAE v4.03) will be used to classify and grade the intensity of adverse events after each treatment cycle. 4. TOI ( Trial outcome index) : TOI will be calculated at baseline and at 6, 12,18,24,36 months in both arms from FACT Head and neck QOL proformas. Increment in TOI of 6 units would be considered significant. The proportion of patients showing an increment in TOI between the 2 arms would be compared by Proportion test. |
| Feasibility | Each year we treat 600 patients with CTRT. Hence if post CTRT each year even if we can recruit 120 patients than in 9 years the recruitment would be over. Hence this study is feasible. |
| Significance | Head and neck cancers are one of the most commonest cancers in the country. Most present locally advanced state. The current options produce unsatisfactory outcomes. Addition of oral metronomic chemotherapy in multiple retrospective studies has been associated with improvement in survival. Hence this study is planning to test this hypothesis. If successful this will be a cost effective and minimally toxic treatment option for patients. |
| Funding | Both intramural and extramural funding options will be explored |
| Risks | Oral metronomic therapy in the study conducted by Pai et al had no grade 3-4 side effects. Even in international studies the incidence of grade 3-4 side effects is below 5%. Hence this seems to be a safe option |

# 4 Background

## 4.1 Outcomes post chemoradiation in locally advanced head & neck cancers

Locally advanced head and neck cancers have poor survivals. The 2 year , 5 year and 10 year overall survival post standard fractionation radiation are 45.6%, 29.3% and 18.3% respectively ( Beitler et al, RTOG 9003).[^1^](https://paperpile.com/c/3p5DlQ/bqQo) The addition of concurrent chemotherapy to radiation leads to an improvement in survival. The 2 year and 5 year overall survival post concurrent chemoradiation are 55 % and 33.7 % in accordance with the MACH-NC analysis .[^2^](https://paperpile.com/c/3p5DlQ/UonL) Even when chemoradiation is done for larynx preservation the overall survivals are at the best modest. The results of RTOG 99-11 revealed a 5 year and 10 year overall survival rate of 55.1% and 27.5%.[^3^](https://paperpile.com/c/3p5DlQ/KRwY)

## 4.2 Failure pattern post radical chemoradiation

The temporal profile of the failure rates reveals that 60% of failures happen in first 2 years.[^2^](https://paperpile.com/c/3p5DlQ/UonL) The sites of failure is predominantly still loco-regional. Around 40% of failures are local, 25% are regional and 35% have both local and regional failures. In these patients 35% of the patients have systemic disease as the site of failure.[^4, 5^](https://paperpile.com/c/3p5DlQ/rA0d+uWju)

## 4.3 Ineffective efforts & need for a new strategy

Strategies to improve upon these outcomes of concurrent chemoradiation have failed. Addition of multiple chemotherapeutic drugs as sensitizer over single agent platinum is not useful.[^2^](https://paperpile.com/c/3p5DlQ/UonL) Similarly addition EGFR receptor inhibitors like cetuximab or panitumumab to concurrent cisplatin and standard fraction radiation too failed to improve outcomes.[^6, 7^](https://paperpile.com/c/3p5DlQ/YoMw+XwPE) The efforts towards modification of fractionation of radiation along with concurrent chemotherapy have also failed.[^8^](https://paperpile.com/c/3p5DlQ/qj7q) Hence at present concurrent cisplatin and standard fraction radiation is considered as the standard of care treatment in locally advanced oropharyngeal cancers and larynx preservation option in locally advanced laryngo-pharyngeal malignancies.[^9, 10^](https://paperpile.com/c/3p5DlQ/avBW+SSSs)

Newer strategies are urgently warranted to improve upon the outcomes in head and neck cancers. Sequencing efforts and integrative genomics have revealed a great deal about the molecular abnormalities that underlie HNSCC. Unfortunately, they have also demonstrated marked heterogeneity, which will make optimizing therapeutic intervention difficult. In HNSCC, oncogenic mutations are rare and mutations in tumor suppressor genes dominate. Restoring function of a tumor suppressor pharmacologically is difficult.[^11^](https://paperpile.com/c/3p5DlQ/SOv7)

## 4.4 In Vivo data for metronomic

In advanced solid tumors it's proposed that adjuvant treatment with metronomic chemotherapy in preclinical mouse models limits metastatic spread.[^12^](https://paperpile.com/c/3p5DlQ/kaGd) A considerable percentage of HNSCC overexpress anti-apoptotic Bcl-2 proteins and that high levels of Bcl-2 correlates with resistance to platinum-based chemotherapy and thus with a poor prognosis.[^13^](https://paperpile.com/c/3p5DlQ/tV25) Imai et al. have demonstrated that ( Metronomic chemotherapy) MC, in association with taxotere decreased both tumor mitotic index and microvessel density and increased survival of mice bearing HNSCC xenografts. Authors evaluated the combination AT101/taxotere on the survival of endothelial cells and HNSCC also in vitro. They observed an additive toxicity for endothelial cells and a synergistic toxicity for tumor cells. Authors concluded that, based on these results, HNSCC patients might benefit from metronomic chemotherapy regimen.[^14^](https://paperpile.com/c/3p5DlQ/ZgMr)

## 4.5 Metronomic chemotherapy : Clinical efficacy data in adjuvant setting

In a study by Lin et al cohort consisted 80 patients with advanced head and neck cancer treated between January 2003 and December 2007. Half of the patients received oral UFUR as metronomic adjuvant chemotherapy, while the other half received no treatment.The disease-free survival rates at 4 years were 84.6% with oral UFUR treatment and 60.9% without UFUR therapy (P = 0.02). The authors concluded that metronomic adjuvant chemotherapy regimen seems a promising option.[^15^](https://paperpile.com/c/3p5DlQ/SBHF)

Similar to this Oral metronomic chemotherapy (OMCT) consisting of methotrexate and celecoxib has shown promise in Perioperative and adjuvant setting in TMH too. Treatment of locally advanced head and neck cancers with adjuvant metronomic chemotherapy post surgery and RT has shown to decrease failure rates and tends to improve upon the survival. In a retrospective review done by Pai et al of advanced T3/T4 head and neck cancers adjuvant OMCT improved the disease free survival by 14.9%. OMCT administered for 18 months seems to have potential for improving outcomes in locally advanced head and neck cancers post radical chemoradiation .[^16^](https://paperpile.com/c/3p5DlQ/0Afq)

In a retrospective review of fifty two head and neck cancer patients done by Furusaka et al metronomic S1 was given for 2 years as adjuvant. Forty-three patients (82.7%) were able to complete the chemotherapy for 2 years without dose reduction. The 3-year disease-free survival rate and 3-year survival rate were 82.6% and 94.0%, respectively.[^17^](https://paperpile.com/c/3p5DlQ/Pw7r)

Similarly in advanced nasopharyngeal cancers too MCT has been evaluated post comple treatment.The study population consisted of 625 NPC patients with available pEBV ( plasma EBV ) DNA levels before and aftertreatment. Eighty-five patients with persistently detectable pEBV DNA after 1 week of completing radiation therapy were eligible for this retrospective study. Of the 85 patients, 33 were administered adjuvant chemotherapy consisting of oral tegafur-uracil (2 capsules twice daily) for 12 months with (n=4) or without (n=29) preceding intravenous chemotherapy of mitomycin-C, epirubicin, and cisplatin. The remaining 52 patients who did not receive adjuvant chemotherapy served as the control group. The adjuvant oral metronomic chemotherapy (OMCT) has been associated with a remarkable improvement in overall survival at 5 years from 27.6% to 71.6%.[^18^](https://paperpile.com/c/3p5DlQ/XaIp)

## 4.6 Toxicity profile of metronomic therapy when used as adjuvant

Adjuvant OMCT has been found to be safe and tolerable. There were no grade 3-4 toxicity associated with OMCT in the study reported by Pai et al . In the study reported by Furusuka et al only two patients (3.8%) had of grade 3- hematological toxicity was noted. In the report of Twu et al only 1 incident of transient of transient leucopenia was seen 93.3%) .

# 5 Rationale for the study

OMCT seems to improve survival when administered as adjuvant treatment in locally advanced head and neck cancers. This therapy is associated with no major grade 2-4 toxicity. Hence we have planned this study to assess the efficacy of adjuvant OMCT (consisting of weekly methotrexate and celecoxib) in locally advanced head and neck cancers post radical chemoradiation.

# 6 Aims & Objectives

## 6.1 Aim

To study whether adjuvant treatment with metronomic chemotherapy leads to a betterment in outcome, in locally advanced head and neck cancers post radical chemoradiation

## 6.2 Objective

### 6.2.1 Primary objective

To compare the 3 year overall survival of adjuvant metronomic chemotherapy with that of observation in patients with locally advanced head and neck cancers post radical chemoradiation .

The primary endpoint would be 3 year overall survival.

### 6.2.2 Secondary objectives

1. To compare the 3 year progression free survival of adjuvant metronomic chemotherapy with that of observation in patients with locally advanced head and neck cancers post radical chemoradiation . The secondary endpoint would be 3 year progression free survival.
2. To compare the grade 3-4 toxicity between adjuvant metronomic chemotherapy and observation
3. To compare the 6,12,18,24 & 36 months QOL scores between the 2 arms

# 7 Hypothesis & trial design

Adjuvant metronomic chemotherapy will result in a 6.5% absolute improvement in 3-year OS rate, which would be considered a clinically meaningful increase .

This will be a 2 arm, parallel design, open label, superiority, explantory phase III randomized controlled trial with multiple interim analysis.

# 8 Study setting

The proposed study will be conducted at head and neck medical oncology department of TMC. Both patients from TMH and ACTREC would be recruited.

# 9 Eligibility criteria

## 9.1 Inclusion criteria

1. Participants must be post radical chemoradiation for a histologically confirmed stage III-IV squamous cell cancers of the head and neck region.
2. Participants must have no gross residual disease post of completion of CTRT
   1. Assessment for same would be done in accordance with institutional standards at a visit post 10-12 weeks of completion of CTRT by PET CT
3. Participants must have malignancy arising from one of the following sites pharynx ( inclusive of oropharynx, hypopharynx) or larynx ( inclusive of supraglottis, glottis and subglottis).
4. Age : Any age above 18 years. No maximum age.
5. ECOG performance status ≤2
6. Participants must have normal organ and marrow function as defined below:
   1. Leukocytes ≥3,000/mcL
   2. Platelets ≥100,000/mcL
   3. Total bilirubin < 1.5 × institutional upper limit of normal
   4. AST(SGOT)/ALT(SGPT) ≤2.5 × institutional upper limit of normal
   5. Calculated Creatinine clearance > 30 ml/min
7. The effects of oral metronomic chemotherapy ( inclusive of methotrexate) on the developing human fetus are teratogenic. Hence women of childbearing potential and men must agree to use adequate contraception (hormonal or barrier method of birth control; abstinence) prior to study entry and for the duration of study participation. Should a woman become pregnant or suspect she is pregnant while she or her partner is participating in this study, she should inform her treating physician immediately. Men treated or enrolled on this protocol must also agree to use adequate contraception prior to the study, for the duration of study participation, and 6 months after completion of protocol.
8. Both men and women of all races and ethnic groups are eligible for this trial.
9. Willing and able to comply with all study requirements, including treatment (able to swallow tablets), able to be followed up at regular intervals and/or nature of required assessments (e.g. able to have IV contrast if this is required for tumour assessments)
10. Ability to understand and the willingness to sign a written informed consent document

## 9.2 Exclusion Criteria

1. Participants who are receiving any other investigational agents.
2. Primary sites of malignancy in oral cavity or major salivary gland or nasopharynx or skin
3. More than Six months post completion of CTRT
4. Patients with QTc prolongation defined as QTc interval greater than 480 ms in view of risk of sudden cardiac death associated with use of celecoxib.
5. Patients receiving methotrexate for other indications not limited to rheumatoid arthritis
6. Patients who had received long term Cox-2 inhibitors ( more than 3 month continuous usage) will be excluded
7. History of allergic reactions attributed to compounds of similar chemical or biologic composition to any agents used in study.
8. Uncontrolled intercurrent illness including, but not limited to, hypertension, tuberculosis, diabetes, ongoing or active infection, symptomatic congestive heart failure, unstable angina pectoris, cardiac arrhythmia, renal failure (on dialysis), active gastrointestinal bleeding, cerebrovascular accidents, inflammatory bowel disease, known hyperkalemia ( CTCAE version 4.02 grade 3 or above which is persistent over 1 week) or psychiatric illness/social situations that would limit compliance with study requirements.
9. Pregnant women and breastfeeding women are excluded from this study because celecoxib / erlotinib and methotrexate are agents with the potential for teratogenic or abortifacient effects. Because there is an unknown but potential risk for adverse events in nursing infants. These potential risks may also apply to other agents used in this study.
10. HIV-positive, Hepatitis B and C seropositive patients are excluded from this study.
11. Patients with previous history of other cancers within last 2 years

# 10 Interventions

## 10.1 Registration

Subjects must meet all of the inclusion criteria and none of the exclusion criteria to be eligible for this trial/study. There will be no exceptions made to these eligibility requirements at the time of registration.

Subjects must be registered before starting study treatment. Treatment should be planned to start within 30 days after registration.Once the registration process has been completed, the subject will be assigned a subject study number, and written confirmation of registration will be provided to the site. Individuals only be registered once in this trial. Following which the patient would be randomize. The randomization procedure is explained in “ Allocation of arm” section.

## 10.2 Arm A

In arm A **observation** would be done. The schedule of observation will be in accordance with the institutional follow up protocol which is as below

**First year** : Patient would be followed up at 3 monthly intervals. Clinical history and examination would be done. Blood investigations like complete hemogram, renal function tests and TSH would be done. FACIT QOL H&N would be filled at baseline,6 and at 12 months. The blood of patients of 10 ml would be collected at baseline, first follow up and at progression in both arms for future research purpose

**Second year** : Patient would be followed up at 3 monthly intervals. Clinical history and examination would be done. Blood investigations like complete hemogram, renal function tests and TSH would be done sos. FACIT QOL H&N would be filled at 18 & 24 months

**Third year-Fifth year** : Patient would be followed up at 6 monthly intervals. Clinical history and examination would be done. Blood investigations like complete hemogram, renal function tests and TSH would be done sos.

**Post fifth year** : Patient would be followed up at 1 year intervals. Clinical history and examination would be done. Blood investigations like complete hemogram, renal function tests and TSH would be done SOS.

## 10.3 Arm B

In arm B **adjuvant metronomic** would be administered.

### 10.3.1 Schedule

Metronomic chemotherapy is the study intervention in this trial. The metronomic chemotherapy constitutes of methotrexate and celecoxib.

Oral methotrexate tablet 15 mg/m2 will be self administered weekly ie on D1,D8, D15 and D22 of every 28 day cycle.

Capsule celecoxib would will be self administered twice daily in a dose of 200 mg BID continuously .

The combination will be continued to a maximum of such 18 cycles; unless prohibitive toxicity or disease progression occurs prior to it. The schedule of visits of these patients would be similar to that in observation arm. Except that ECG and LFT would be done at each visit.

### 10.3.2 Administration

Lowest strength Methotrexate tablet is available as a 2.5 mg tablet. The recommended dose should be rounded to the nearest lower multiple of 2.5 mg. All tablets should be taken with at least half a glass of water one hour prior to or two hours post food. Capsule celecoxib should be taken twice daily 12 hours apart one hour post meal.

### 10.3.3 Dose modifications

Instructions for treatment delays and dose modifications for adverse events are specified below. Adverse events will be graded according to National Cancer Institute ,Common Terminology Criteria for Adverse Events (CTCAE) version 4.03. In general, treatment should be withheld during adverse events of severity G3-4, and not restarted until the adverse event has resolved to G0-1, at the investigator’s discretion. Day 1 treatment may be delayed for a maximum of 14 days. If the adverse event has not resolved to G0-1 after delaying day 1 treatment for 14 days, then study treatment should be discontinued. Treatment should not be delayed or modified for alopecia of any grade.

Specified dose reductions apply to all subsequent doses of study drug. If a patient experiences several adverse events with differing recommendations, then the modification that results in the longest delay and lowest dose should be used. Dose escalations or dose re-escalations after reductions for adverse events are prohibited.

#### 10.3.3.1 Dose modification for hematological toxicity

| **PARAMETER** | **TIMING** | **RESULT OR PROBLEM** | **CTCAE GRADE** | **ACTION WITH STUDY TREATMENT**  **(Methotrexate unless otherwise specified)** | | | | |
| --- | --- | --- | --- | --- | --- | --- | --- | --- |
| Neutrophils  (x10^9^/L) | Previous cycle | Febrile neutropenia, or infection with neutropenia | G4 | Delay D1 until resolved AND  reduce dose of methotrexate by 20% | | | | |
|  |  |  |  |  |  |  |  |  |
|  | D1 | 1.0-1.5 | G2 | Delay D1 until ANC is >1.5 AND  If delay > 7 days but < 15days, reduce doses by 20% level.  If delay >15 days then discontinue. | | | | |
|  |  | <1.0 | G3-4 | Delay D1 until ANC is >1.5 AND  reduce doses by 20% level. | | | | |
| Platelets  (x10^9^/L) | Previous cycle | < 25 or bleeding | G4 | Delay D1 until resolved AND  reduce methotrexate doses by 20% | | | | |
|  | D1 | <100 | G1-4 | Delay D1 until platelet count is > 99 x10^9^/L AND  If delay > 7 d but <15 days reduce doses by 20% level.  If delay > 15d then discontinue. | | | | |
| Haemoglobin  (g/L) | First occasion | <80 | G2 | Transfuse to Hb > 80 g/L AND  treat as scheduled. | | | | |
|  | Second occasion | <80 | G2 | Transfuse to Hb >80, treat as scheduled, AND reduce doses of methotrexate by 20% | | | | |
| Day 1 treatment may be delayed for a maximum of 14 days. If the adverse event has not resolved to G0-1 after delaying day 1 treatment for 14 days, then chemotherapy should be discontinued.  **- No dose reduction in celecoxib recommended. However, if chemotherapy is discontinued then celecoxib should also be discontinued. | | | | |  |  |  |  |

Table 1 : Dose modification in hematological events

#### 10.3.3.2 Dose modifications for other adverse events

| System | CTCAE term | CTCAE grade | Other details | Actions with drugs | Other actions |
| --- | --- | --- | --- | --- | --- |
| Hepatic | Bilirubin | Grade 3-4 | >3.0 ULN | Discontinue methotrexate |  |
| Hepatic | AST/ALT | Grade 3-4 | >5.0 x ULN AND  > 2 x baseline | Discontinue methotrexate |  |
| Renal | Creatinine | G2-4 | >1.5 x baseline AND  > 1.5 x ULN | Withhold both methotrexate and celecoxib until <1.5 x baseline  AND then restart at 20% lower dose |  |
| Infection | Infection | G3-4 |  | Delay until G0-1  Restart when G0-1  Reduce doses by 20% of methotrexate |  |
| GI | Nausea | G3-4 |  | Delay until G0-1  Restart when G0-1  Reduce doses by 20% of methotrexate |  |
| GI | Vomiting | G3-4 |  | Delay until G0-1  Restart when G0-1  Reduce doses by 20% of methotrexate |  |
| GI | Diarrhea | G3-4 |  | Delay until G0-1  Restart when G0-1  Reduce doses by 20% of methotrexate |  |
| GI | Mucositis | G3-4 |  | Delay until G0-1  Restart when G0-1  Reduce doses by 20% of methotrexate |  |
| Cardiovascular | Myocardial infarction | G3-4 |  | Discontinue methotrexate 7 celecoxib |  |
| Vascular | Thrombo-embolic event | G3-4 | Venous ( DVT or PTE) | Delay both methotrexate & celecoxib until adequately treated  Restart at physician discretion | Anticoagulate with heparin, not warfarin, whilst on study drug |
| Vascular | Thrombo-embolic event | G3-4 | Arterial | Discontinue both methotrexate and celecoxib |  |
| Skin | Various | G3-4 |  | Delay methotrexate and celecoxib until G0-1  Restart when G0-1  Reduce subsequent doses 20% |  |

Table 2: Dose modification for non hematological toxicity

### 10.3.4 Start of a new cycle

Re-treatment on day 1 will require: haemoglobin > 8 mg/dl, ANC count > 1500/mm3, platelet count > 1 lakh/mm3, creatinine clearance rate >30 ml min, and resolution of all non haematological toxicities (except alopecia and fatigue) to baseline or less than grade 1.

If methotrexate is delayed or withheld and if the cause of the delay does not require an interruption in celecoxib then it will be continued. Subsequently methotrexate can be given on the next weekly scheduled day. The scheduled methotrexate which was omitted remains omitted. The cycle would be considered continued if celecoxib is continued. If both are delayed and the delay is less than 15 days then from the day of restart the remaining cycle would be completed.

### 10.3.5 Concomitant Medications/Treatments

#### 10.3.5.1 Recommended

The following medications and treatments are recommended in this study:

1. Antiemetics if required by the patient. The antiemetics recommended are 5HT3 antagonist with or without prokinetics
2. No other medications or treatments are specifically recommended in this study.

#### 10.3.5.2 Permitted

The following medications and treatments are permitted in this study:Medications for chronic medical comorbidities like diabetes, hypertension.

1. Symptomatic medications like H2 blockers, antacids, antidiarrheal agents and stool softeners

#### 10.3.5.3 Use with caution

The following medications are best avoided whilst subjects are on study drug, and must be used with caution:

1. GM CSF, erythropoietic agents and other hematopoietic agents should not be used to avoid dose reductions
2. Drugs whose metabolism may be affected by study drug eg allopurinol.
3. NSAIDS for analgesia
4. Aspirin upto a dose of 100mg
5. Low molecular weight heparin
6. Proton pump inhibitors

#### 10.3.5.4 Prohibited

The following medications should not be used during this study.

- Drugs like warfarin, aspirin ≥ 325 mg/day, antiplatelet agents.

### 10.3.6 Concomitant medication reporting

Concomitant medications will not be recorded during the study, except for medications used to treat serious adverse events or medications known to interact with the study medications.

List of this medications include

1. NSAIDS

2. Aspirin

3. Warfarin

4. Allopurinol

5. Antiplatelet agents

6. Antidiarrhoeal agents

7. Stool softeners

8. Sulfa drugs

### 10.3.7 Compliance

Subject medication compliance will be determined at each clinic visit by interview of patient & relative and the patient will be counselled appropriately if significant non-compliance is determined.

## 10.4 Treatment discontinuation

Study treatment will be permanently discontinued for any of the following reasons:

1. Progressive disease (PD) is documented by the investigator.
2. Unacceptable toxicity as determined by the patient or site investigator or as defined in Table 1 & 2
3. Delay of treatment for 14 days due to treatment-related adverse events. For delays >14 days due to due to reasons other than treatment-related adverse events. The treatment would be continued. For delay > 21 days due to due to reasons other than treatment-related adverse events. The treatment would be discontinued.
4. The investigator determines that continuation of treatment is not in the patient’s best interest.
5. Occurrence of an exclusion criterion affecting patient safety, e.g. pregnancy or psychiatric illness.
6. Required use of a concomitant treatment that is not permitted, as defined in section of prohibited medications.
7. Failure to comply with the protocol, e.g. repeatedly failing to attend scheduled assessments. If a patient has failed to attend scheduled assessments in the study, the Investigator must determine the reasons and document the circumstances as completely and accurately as possible in the medical records and CRF.
8. The patient declines further study treatment, or withdraws their consent to participate in the study.

The reasons for discontinuing treatment will be documented in the subject’s medical record.

Follow up of subjects who stop study treatment should continue according to this protocol

## 10.5 Subsequent treatment

Treatment after discontinuation of study treatment is at the discretion of the patient’s clinician.

# 11 Outcomes & Statistical measures

## 11.1 Primary outcome

1. 3 year OS : OS will be defined time in days between date of randomization to date of death. Patients alive at their last follow ups would be censored. The 3 year OS would be estimated by Kaplan meier analysis and would be compared between the 2 arms by the log rank test. Cox proportional hazard model would be constructed for calculation of hazard ratio. The model would also be used to see the impact of chemotherapy regimen on 3 year OSin accordance with known prognostic factors. Age ( below or above 70 years), subsite , T stage, N stage, hemoglobin level ( equal to or below 10 g/dl or above it), and PS ( 0-1 versus 2).This information would be depicted in the form of a forest plot.

## 11.2 Secondary outcomes

1. 3 year PFS : PFS will be defined time in days between date of randomization to date of progression or death whichever is earlier. Patients who have not progressed or died at their last follow ups would be censored.The 3 year PFS would be estimated by Kaplan meier analysis and would be compared between the 2 arms by the log rank test. Cox proportional hazard model would be constructed for calculation of hazard ratio. The model would also be used to see the impact of chemotherapy regimen on 3 year progression survival in accordance with known prognostic factors. Age ( below or above 70 years), subsite , T stage, N stage, hemoglobin level ( equal to or below 10 g/dl or above it), and PS ( 0-1 versus 2).This information would be depicted in the form of a forest plot.
2. Toxicity : To compare CTCAE version 4.03 worst grade toxicity between the 2 arms during any period of chemotherapy. Chi- square test or fisher's test would be used for comparison. The NCI Common Terminology Criteria for Adverse Events version 4 (NCI CTCAE v4.03) will be used to classify and grade the intensity of adverse events after each treatment cycle. CTCAE will be used to collect all events regardless of attribution, in order to ensure objective reporting, and in order to report trial data according to accepted international guidelines. The worst toxicity would be recorded. The results would be computed in a tabular form in which the proportion of people having their highest grade of toxicity would be charted.
3. TOI ( Trial outcome index) : TOI will be calculated at baseline and at 6, 12,18,24 & 36months in both arms from FACT Head and neck QOL proformas. Increment in TOI of 6 units would be considered significant. Proportion of patients with significant improvement in TOI will be compared between 2 arms. Proportion test would be used for comparison.

# 12 Participant timelines

| Investigations | Baseline | At schedule visit | Progression |
| --- | --- | --- | --- |
| History | √ | √ | √ |
| Drug allergy | √ | X | X |
| Local examination | √ | √ | √ |
| CECT neck + relevant part | X | * | * |
| PET-CT | √ | X | X |
| Toxicity assessment | X | √ | √ |
| Biopsy / FNA | X | X | √ |
| CBC | √ | √# | √ |
| RFT | √ | √# | √ |
| LFT | √ | √# | √ |
| SE ,Ca, Mg | √ | √# | √ |
| TSH | √ | * | * |
| ECG | √ | X | X |
| b-HCG | √ | X | X |
| FACT -H&N QOL | √ | √ | X |
| Blood sample 10 Ml ( 5 ml EDTA + 5 ml Plain) | √ | √*** | √ |

**Table 3 : Timeline for participants in Arm : A**

***- CECT neck + scan of the relevant part would be done in accordance with the institutional standards.**

**#-Investigations will be done as per institutional standards and sos**

****- FACT H&N QOL would be collected at 6, 12,18,24 & 36 months from start of chemotherapy**

******- Blood sample will be collected only at the first 3 month visit.**

| Investigations | Baseline | D1 of chemotherapy for 18 cycles ( every 3 month in till 18 months) | Post 18 months at schedule visit | Progression |
| --- | --- | --- | --- | --- |
| History | √ | √ | √ | √ |
| Drug allergy | √ | X | X | X |
| Local examination | √ | √ | √ | √ |
| CECT neck + relevant part | X | * | * | * |
| PET-CT | √ | X | X | X |
| Toxicity assessment | X | √ | √ | √ |
| Biopsy / FNA | X | X | X | √ |
| CBC | √ | √ | √# | √ |
| RFT | √ | √ | √# | √ |
| LFT | √ | √ | √# | √ |
| SE ,Ca, Mg | √ | √ | √# | √ |
| TSH | √ | * | * | * |
| ECG | √ | √ | X | X |
| b-HCG | √ | X | X | X |
| FACT -H&N QOL | √ | ** | X | X |
| Blood sample 10 Ml ( 5 ml EDTA + 5 ml Plain) | √ | √*** | X | √ |

**Table 4 :Timeline for participants in Arm : B**

***- CECT neck + scan of the relevant part would be done in accordance with the institutional standards.**

**#-Investigations will be done as per institutional standards and sos**

****- FACT H&N QOL would be collected at 6, 12,18,24 & 36 months from start of chemotherapy**

******- Blood sample will be collected only at the first 3 month visit.**

# 13 Sample size

## 13.1 Rationale for selection of primary endpoint : 3 year OS

The primary endpoint selected for sample size calculation is 3 year overall survival. The reason for this being

1. In Head and neck cancers majority of events happen in 2-3 years post treatment completion
2. Availability of data from our institute, on the 3 year outcomes of PET-CT negative patients post radical CTRT.

The 3 years overall survival post radical CTRT in PET-CT negative patients is 68.8%~ 69%. The 3 year overall survival of 69% or more is obtained from multiple international studies reporting results of CTRT in stage III-IV head and neck cancers.

| Study name | n | Stages included | 3 year OS |
| --- | --- | --- | --- |
| RTOG 0522 | 891 | Stage III & IV head & neck cancers.  Randomly assigned to receive radiation and cisplatin without (arm A) or with (arm B) cetuximab. | Arm A :72.9% *v Arm B:* 75.8%, respectively; *P* = .32) |
| RTOG 0129 | 743 | Patients had **stage III to IV carcinoma of the oral cavity, oropharynx, hypopharynx, or larynx.** Radiation therapy schedules were 70 Gy in 35 fractions over 7 weeks (SFX) or 72 Gy in 42 fractions over 6 weeks (AFX-C). Cisplatin doses were 100 mg/m^2^ once every 3 weeks for two (AFX-C) or three (SFX) cycles. | 8-year survival, 48% *v* 48%). **The 3 year OS was around 70%** in both arms. |
| DeCIDE | 285 | In this phase 3, open-label trial, subjects with **pathologically confirmed SCCHN; N2/N3 disease without metastases**; no prior therapy; KPS ³ 70%; and intact organ function were randomized to CRT alone (CRT arm) [5 days of D (25 mg/m2), F (600 mg/m2), hydroxyurea (500 mg BID), and RT (150 cGy BID) followed by a 9 day break] or to 2 cycles of IC [D (75 mg/m2), P (75 mg/m2), F (750 mg/m2 day 1-5)] followed by the same CRT (IC arm). | **3 year OS was 75% and 73% in CTRT & IC-CTRT** |
| PARADIGM | 145 | Patients were eligible if their tumour was either **unresectable or of low surgical curability on the basis of advanced tumour stage (3 or 4) or regional-node stage (2 or 3, except T1N2), or if they were a candidate for organ preservation**. Patients were randomly assigned (in a 1:1 ratio) to receive either induction chemotherapy with three cycles of TPF followed by concurrent chemoradiotherapy with either docetaxel or carboplatin or concurrent chemoradiotherapy alone with two cycles of bolus cisplatin. | **3-year overall survival was 73% (95% CI 60-82) in the induction therapy followed by chemoradiotherapy group and 78% (66-86) in the chemoradiotherapy alone group (hazard ratio 1·09, 95% CI 0·59-2·03; p=0·77)** |
| **Mehanna et al** | 564 | In this prospective, randomized, controlled trial, the authors assessed the noninferiority of positron-emission tomography–computed tomography (PET-CT)–guided surveillance (performed 12 weeks after the end of chemoradiotherapy, with neck dissection performed only if PET-CT showed an incomplete or equivocal response) to planned neck dissection in patients with stage N2 or N3 disease. | The 2-year overall survival rate was 84.9% (95% confidence interval [CI], 80.7 to 89.1) in the surveillance group and 81.5% (95% CI, 76.9 to 86.3) in the planned-surgery group. **The 3 year OS is well above 70%.** |

Table 5 : Studies supporting 3 year OS of 69-70% in stage III-IV head and neck cancers post CTRT.

## 13.2 Rationale for interim analysis

The survival benefit shown with adjuvant metronomic studies is limited sample size cohort studies. Hence as the investigators plan to do a study which is powered for an absolute survival improvement of 6% at 3 year in OS.It was decided to have a multiple interim analysis.

## 13.3 Sample size justification

### 13.3.1 Sample size

The primary endpoint for is 3 year OS. The study assumes a 3 year OS in standard arm of 69%. With Type I error of 5% , Type II error of 20%, for a hazard ratio of 0.77, with study duration of recruitment of 9 years, study follow up of 3 years and 5 % lost to follow up the sample size was calculated using fixed design, two-arm trial with time-to-event

outcome (Lachin and Foulkes, 1986) in Rstudio version 3.2.1. The sample size required was 950 patients with analysis done at 485 events. However in view of the large sample size 3 interim analysis were included in this study using Group sequential design sample size with Hwang-Shih-DeCani spending function for time-to-event outcome events. The final **sample size was 1038 patients**. The analysis plan below shows events at each analysis with the decision rules.

**Decision rules**

Upper bound spending computations assume trial continues if lower bound is crossed.

----Lower bounds---- ----Upper bounds-----

Analysis N Z Nominal p Spend+ Z Nominal p Spend++

1st interim 133 -0.75 0.2275 0.0203 2.95 0.0016 0.0016

2nd interim 265 0.13 0.5509 0.0335 2.58 0.0049 0.0044

3rd interim 398 0.91 0.8180 0.0552 2.17 0.0151 0.0118

Final analysis 530 1.69 0.9548 0.0910 1.69 0.0452 0.0322

Total 0.2000 0.0500

+ lower bound beta spending (under H1):

Hwang-Shih-DeCani spending function with gamma = -2.

++ alpha spending:

Hwang-Shih-DeCani spending function with gamma = -4.

Boundary crossing probabilities and expected sample size

assume any cross stops the trial

# 14 Recruitment

All patients post radical chemoradiation in Head and neck medical oncology OPD ( general, private or at ACTREC) would be informed about the study and if they are willing would be screened for the same.

# 15 Allocation of arm

## 15.1 Sequence generation

Stratified randomization would be done. The stratification would be for site of tumor (oropharynx versus larynx versus hypopharynx), T stage ( T1-2, T3 , T4) and N stage ( N0, N1, N2-N3). The randomization sheet would be computer generated. Twenty seven sheets would be generated.

## 15.2 Allocation concealment mechanism

These sheets would be maintained within the protocol room. The trial PI, Co-I and trial coordinators won't have access to it. One of the trial coordinators of other head and neck medical oncology study would be entitled with its responsibility.

## 15.3 Implementation

Investigators or trial coordinators would email the person incharge about the patient's details ( site of tumor, T stage and N stage). The person incharge of the randomization sheet would issue a randomization number and the arm.

# 16 Data collection methods

The data of the study would be collected in a pre designed case record form. The data will be filled in excel sheets and then would be transferred in SPSS and/or R studio for requisite analysis.

# 17 Data monitoring

The institutional data monitoring and safety board (DSMB) will be responsible for oversight of the data.

# 18 Harms

An ADVERSE EVENT (AE) is any untoward medical occurrence in a patient or clinical investigational subject administered a pharmaceutical product and which does not necessarily have a causal relationship with this treatment. An AE can therefore be any unfavourable or unintended sign (including an abnormal laboratory finding), symptom, or disease temporally associated with the use of a medicinal investigational product, whether or not considered related to the medicinal product (see below).

Adverse events include the following

All suspected adverse drug or device reactions

1. All reactions from drug or device – overdose, abuse, withdrawal, sensitivity, toxicity or failure of expected pharmacological action (if appropriate)
2. Apparently unrelated illnesses, including the worsening (severity, frequency) of pre-existing illnesses
3. Injury or accidents.
4. Abnormalities in physiological testing or physical examination that require clinical intervention or further investigation (beyond ordering a repeat examination)
5. Laboratory abnormalities that require clinical intervention or further investigation (beyond ordering a laboratory test)
6. Any untoward event that occurs after the protocol-specified reporting period which the Investigator believes may be related to the drug or device.

**AEs are not required to be reported unless they meet SAE criteria.**

A SERIOUS ADVERSE EVENT (SAE) is any untoward medical occurrence that at any dose:

1. results in death,
2. is life-threatening (i.e. the subject is at risk of death at the time of the event),
3. requires inpatient hospitalisation or prolongation of existing hospitalisation,
4. results in persistent or significant disability or incapacity,
5. is a congenital anomaly/birth defect,
6. other important medical events which, in the opinion of the investigator, are likely to become serious if untreated, or as defined in the protocol

NOTES:

(i) The term “life-threatening” in the definition of “serious” refers to an event in which the patient was at risk of death at the time of the event; it does not refer to an event which hypothetically might have caused death if it were more severe.

(ii) Important medical events which may not be immediately life-threatening or result in death or hospitalization but which may jeopardize the patient or may require intervention to prevent one of the listed outcomes in the definition above should also be considered serious.

# 19 Auditing

The trial data will be audited at 6 monthly intervals to monitor the participant enrolment, consent, eligibility, and allocation to study groups; adherence to trial interventions and policies to protect participants, including reporting of harms; and completeness, accuracy, and timeliness of data collection. An independent auditing body will be tasked with this purposed which has been identified by the data safety and monitoring body (DSMB).

# 20 Research ethics approval

This protocol and the template informed consent forms contained in Appendix will be reviewed and approved by the institutional IRB with respect to scientific content and compliance with applicable research and human subjects regulations. The protocol, site-specific informed consent forms (local language and English versions), participant education and recruitment materials, and other requested documents—and any subsequent modifications — also will be reviewed and approved by the IRB. Subsequent to initial review and approval, the IRB will review the protocol at least annually. The Investigator will make safety and progress reports to the IRB at 12 monthly intervals and within three months of study termination or completion. These reports will include the total number of participants enrolled and summaries of each DSMB [data safety and monitoring board] review of safety and/or efficacy.

# 21 Protocol amendments

Any modifications to the protocol which may impact on the conduct of the study, potential benefit of the patient or may affect patient safety, including changes of study objectives, study design, patient population, sample sizes, study procedures, or significant administrative aspects will require a formal amendment to the protocol. Any and all such amendments will be communicated to the institutional IRB for review and approval. Administrative changes of the protocol are minor corrections and/or clarifications that have no effect on the way the study is to be conducted. These may be communicated to the IRB at the investigator's’ discretion.

# 22 Consent

Patients will be given the patient information sheet by the trial investigators / nurses. The purpose and reasons behind the study will be communicated to the patient. All patients will be provided with a copy of the written informed consent as well as the patient information sheet. Consent will be recorded on as per institutional IRB guidelines.

# 23 Confidentiality

All study-related information will be stored securely at the study site. All participant information will be stored in locked file cabinets in areas with limited access. All laboratory specimens, reports, data collection, process, and administrative forms will be identified by a coded ID [identification] number only to maintain participant confidentiality. All records that contain names or other personal identifiers, such as locator forms and informed consent forms, will be stored separately from study records identified by code number. All local databases will be secured with password-protected access systems. Forms, lists, logbooks, appointment books, and any other listings that link participant ID numbers to other identifying information will be stored in a separate, locked file in an area with limited access.

# 24 Access to data

TMH : The Principal Investigator and Co investigators will be given access to the data sets. Project data sets will be housed on the project specific database created for the study, and it will be password protected.

Other centers : The Data Management Coordinating Center will oversee the intra-study data sharing process, with input from the Data Management Subcommittee. All Principal Investigators will be given access to the cleaned data sets. Project data sets will be housed on the project specific database created for the study, and all data sets will be password protected.

# 25 Ancillary & post trial care

Patients metronomic treatment and supportive care cost would be beared by the study budget . Patients will be treated further in accordance with institutional guidelines. In addition in the event of intolerable side effects or disease progression palliative radiotherapy or chemotherapy will be used for their treatment if feasible. These costs will not be reimbursed for the patient as they are standard of care for such patients. Patients will be treated for trial related adverse events in the hospital. Compensation for adverse events will be provided in accordance with institutional standards.

# 26 Dissemination policy

The trial results will be submitted for publication in international peer reviewed journals and presented at national/international conferences. No publication restrictions will be imposed by trial sponsors. All co investigators will be given authorship on the manuscript as well as on any abstract that are reported in national/international conferences. The principal investigator will take either the role of the corresponding author or the first author positions as decided mutually among the investigators. Authorship criteria for other publications stemming from the study will be decided as per the relative contributions and existing institutional guidelines. The contribution of all investigators will be acknowledged in such manuscripts if they are not eligible for authorship. In addition the grant giving organizations will also be acknowledged in all publications.

# 27 References

[**1**. Beitler JJ, Zhang Q, Fu KK, et al: Final results of local-regional control and late toxicity of RTOG 9003: a randomized trial of altered fractionation radiation for locally advanced head and neck cancer. Int J Radiat Oncol Biol Phys 89:13–20, 2014](http://paperpile.com/b/3p5DlQ/bqQo)

[**2**. Pignon J-P, le Maître A, Maillard E, et al: Meta-analysis of chemotherapy in head and neck cancer (MACH-NC): an update on 93 randomised trials and 17,346 patients. Radiother Oncol 92:4–14, 2009](http://paperpile.com/b/3p5DlQ/UonL)

[**3**. Forastiere AA, Zhang Q, Weber RS, et al: Long-term results of RTOG 91-11: a comparison of three nonsurgical treatment strategies to preserve the larynx in patients with locally advanced larynx cancer. J Clin Oncol 31:845–852, 2013](http://paperpile.com/b/3p5DlQ/KRwY)

[**4**. Oksuz DC, Prestwich RJ, Carey B, et al: Recurrence patterns of locally advanced head and neck squamous cell carcinoma after 3D conformal (chemo)-radiotherapy. Radiat Oncol 6:54, 2011](http://paperpile.com/b/3p5DlQ/rA0d)

[**5**. Bayman E, Prestwich RJD, Speight R, et al: Patterns of failure after intensity-modulated radiotherapy in head and neck squamous cell carcinoma using compartmental clinical target volume delineation. Clin Oncol 26:636–642, 2014](http://paperpile.com/b/3p5DlQ/uWju)

[**6**. Ang KK, Zhang Q, Rosenthal DI, et al: Randomized phase III trial of concurrent accelerated radiation plus cisplatin with or without cetuximab for stage III to IV head and neck carcinoma: RTOG 0522. J Clin Oncol 32:2940–2950, 2014](http://paperpile.com/b/3p5DlQ/YoMw)

[**7**. Mesía R, Henke M, Fortin A, et al: Chemoradiotherapy with or without panitumumab in patients with unresected, locally advanced squamous-cell carcinoma of the head and neck (CONCERT-1): a randomised, controlled, open-label phase 2 trial. Lancet Oncol 16:208–220, 2015](http://paperpile.com/b/3p5DlQ/XwPE)

[**8**. Nguyen-Tan PF, Zhang Q, Ang KK, et al: Randomized phase III trial to test accelerated versus standard fractionation in combination with concurrent cisplatin for head and neck carcinomas in the Radiation Therapy Oncology Group 0129 trial: long-term report of efficacy and toxicity. J Clin Oncol 32:3858–3866, 2014](http://paperpile.com/b/3p5DlQ/qj7q)

[**9**. Pfister DG, Ang K-K, Brizel DM, et al: Head and neck cancers, version 2.2013. Featured updates to the NCCN guidelines. J Natl Compr Canc Netw 11:917–923, 2013](http://paperpile.com/b/3p5DlQ/avBW)

[**10**. Grégoire V, Lefebvre J-L, Licitra L, et al: Squamous cell carcinoma of the head and neck: EHNS–ESMO–ESTRO Clinical Practice Guidelines for diagnosis, treatment and follow-up. Ann Oncol 21:v184–v186, 2010](http://paperpile.com/b/3p5DlQ/SSSs)

[**11**. Riaz N, Morris LG, Lee W, et al: Unraveling the molecular genetics of head and neck cancer through genome-wide approaches. Genes Dis 1:75–86, 2014](http://paperpile.com/b/3p5DlQ/SOv7)

[**12**. Srivastava K, Hu J, Korn C, et al: Postsurgical adjuvant tumor therapy by combining anti-angiopoietin-2 and metronomic chemotherapy limits metastatic growth. Cancer Cell 26:880–895, 2014](http://paperpile.com/b/3p5DlQ/kaGd)

[**13**. De Felice F, Musio D, Tombolini V: Head and neck cancer: metronomic chemotherapy. BMC Cancer 15:677, 2015](http://paperpile.com/b/3p5DlQ/tV25)

[**14**. Imai A, Zeitlin BD, Visioli F, et al: Metronomic dosing of BH3 mimetic small molecule yields robust antiangiogenic and antitumor effects. Cancer Res 72:716–725, 2012](http://paperpile.com/b/3p5DlQ/ZgMr)

[**15**. Lin J-S, Cheng C-Y, Liu C-J: Oral uracil and tegafur as postoperative adjuvant metronomic chemotherapy in patients with advanced oral squamous cell carcinoma [Internet]. Journal of Dental Sciences Available from:](http://paperpile.com/b/3p5DlQ/SBHF) <http://www.sciencedirect.com/science/article/pii/S1991790215000574>

[**16**. Pai PS, Vaidya AD, Prabhash K, et al: Oral metronomic scheduling of anticancer therapy-based treatment compared to existing standard of care in locally advanced oral squamous cell cancers: A matched-pair analysis. Indian J Cancer 50:135–141, 2013](http://paperpile.com/b/3p5DlQ/0Afq)

[**17**. Furusaka T, Tanaka A, Matsuda H, et al: Consecutive daily low-dose S-1 adjuvant chemotherapy after radical treatment for squamous cell carcinoma in head and neck cancer. Acta Otolaryngol 131:1099–1103, 2011](http://paperpile.com/b/3p5DlQ/Pw7r)

[**18**. Twu C-W, Wang W-Y, Chen C-C, et al: Metronomic adjuvant chemotherapy improves treatment outcome in nasopharyngeal carcinoma patients with postradiation persistently detectable plasma Epstein-Barr virus deoxyribonucleic acid. Int J Radiat Oncol Biol Phys 89:21–29, 2014](http://paperpile.com/b/3p5DlQ/XaIp)
